# Supplementary material for: Elevated genetic risk for multiple sclerosis emerged in steppe pastoralist populations
Source: Nature. 2024 Jan 10;625(7994):321–8. doi: 10.1038/s41586-023-06618-z (PMC10781639; doi:10.1038/s41586-023-06618-z)
Supplement: Supplementary file 2 — Reporting Summary [file 41586_2023_6618_MOESM2_ESM.pdf]

nature portfolio

Corresponding author(s): Eske Willerslev, Lars Fugger, Dan Lawson, Astrid Iversen

Last updated by author(s): Jul 20, 2023

Reporting Summary

Nature Portfolio wishes to improve the reproducibility of the work that we publish. This form provides structure for consistency and transparency in reporting. For further information on Nature Portfolio policies, see our Editorial Policies and the Editorial Policy Checklist.

Statistics

For all statistical analyses, confirm that the following items are present in the figure legend, table legend, main text, or Methods section.

n/a	Confirmed	
☐	☒	The exact sample size (*n*) for each experimental group/condition, given as a discrete number and unit of measurement
☐	☒	A statement on whether measurements were taken from distinct samples or whether the same sample was measured repeatedly
☐	☒	The statistical test(s) used AND whether they are one- or two-sided<br>*Only common tests should be described solely by name; describe more complex techniques in the Methods section.*
☐	☒	A description of all covariates tested
☐	☒	A description of any assumptions or corrections, such as tests of normality and adjustment for multiple comparisons
☐	☒	A full description of the statistical parameters including central tendency (e.g. means) or other basic estimates (e.g. regression coefficient) AND variation (e.g. standard deviation) or associated estimates of uncertainty (e.g. confidence intervals)
☐	☒	For null hypothesis testing, the test statistic (e.g. *F*, *t*, *r*) with confidence intervals, effect sizes, degrees of freedom and *P* value noted<br>*Give P values as exact values whenever suitable.*
☒	☐	For Bayesian analysis, information on the choice of priors and Markov chain Monte Carlo settings
☒	☐	For hierarchical and complex designs, identification of the appropriate level for tests and full reporting of outcomes
☐	☒	Estimates of effect sizes (e.g. Cohen's *d*, Pearson's *r*), indicating how they were calculated

Our web collection on statistics for biologists contains articles on many of the points above.

Software and code

Policy information about availability of computer code

Data collection

GLIMPSE
BCL Convert
AdapterRemoval (2.2.4)
BWA (0.7.17)
Picard MarkDuplicates (2.18.26)
mapDamage2.0
ContamMix
ANGSD (0.931)

Data analysis	READ<br>HaploGrep2<br>PLINK2<br>ADMIXTURE<br>qpAdm<br>Chromopainter<br>The modified version of CLUES used in this study is available from https://github.com/standard-aaron/clues. The pipeline and conda environment necessary to replicate the analysis of allele frequency trajectories and polygenic selection in Supplementary Note 6 are available on Github at https://github.com/ekirving/ms_paper. The code to create Ancestry Anomaly scores based on Chromosome painting is on Github at https://github.com/danjlawson/ms_paper. The code to compute LDA and LDA score is available on Github at https://github.com/YaolingYang/LDAandLDAscore. The code for HTRX is on Github at https://github.com/YaolingYang/HTRX. The code for ARS calculation is on Github at https://github.com/will-camb/ms_paper.

For manuscripts utilizing custom algorithms or software that are central to the research but not yet described in published literature, software must be made available to editors and reviewers. We strongly encourage code deposition in a community repository (e.g. GitHub). See the Nature Portfolio guidelines for submitting code & software for further information.

Data

Policy information about availability of data

All manuscripts must include a data availability statement. This statement should provide the following information, where applicable:

- Accession codes, unique identifiers, or web links for publicly available datasets
- A description of any restrictions on data availability
- For clinical datasets or third party data, please ensure that the statement adheres to our policy

All collapsed and paired-end sequence data for novel samples sequenced in this study will be made publicly available on the European Nucleotide Archive, together with trimmed sequence alignment map files, aligned using human build GRCh37. Previously published ancient genomic data used in this study are detailed in ST13, and are all already publicly available.

The UK Biobank is a public resource open to approved researchers. More information at https://www.ukbiobank.ac.uk/
The 1000 Genomes resource is publicly available. More information at https://www.internationalgenome.org/

Research involving human participants, their data, or biological material

Policy information about studies with human participants or human data. See also policy information about sex, gender (identity/presentation), and sexual orientation and race, ethnicity and racism.

Reporting on sex and gender	Sex was assigned based on sex chromosomes. Sex-specific results were not calculated.
Reporting on race, ethnicity, or other socially relevant groupings	Reporting was restricted to a self-identified 'white British' cohort, with PCA outliers removed (details in Bycroft et al., 2018).
Population characteristics	N/A
Recruitment	N/A
Ethics oversight	Use of the UK Biobank resource was approved in 2020.

Note that full information on the approval of the study protocol must also be provided in the manuscript.

Field-specific reporting

Please select the one below that is the best fit for your research. If you are not sure, read the appropriate sections before making your selection.

☐ Life sciences ☐ Behavioural & social sciences ☒ Ecological, evolutionary & environmental sciences

For a reference copy of the document with all sections, see nature.com/documents/nr-reporting-summary-flat.pdf

Ecological, evolutionary & environmental sciences study design

All studies must disclose on these points even when the disclosure is negative.

Study description	This study uses data from ancient and modern individuals to estimate ancestral contributions to risk for multiple sclerosis and rheumatoid arthritis, and test for signals of selection.
Research sample	Modern samples were from the UK Biobank and 1000 Genomes project. Ancient samples were previously published, and 86 new samples published here for the first time.

Sampling strategy	86 samples is enough to capture much of the genetic variation present in Medieval Denmark. It also brings this population to a similar sampling size as other ancient groups.
Data collection	The ancient sampling procedure is described in the SI. Sequencing is described here:<br>SSequencing data was generated from a total of 86 Medieval samples (ST1), using semi-automated laboratory procedures. Laboratory work on aDNA was conducted in the dedicated ancient DNA clean-room facilities at the Lundbeck Foundation GeoGenetics Centre (Globe Institute, University of Copenhagen).<br><br>In brief, two parallel sub-samples of <150 mg were obtained from human skeletal material and demineralized as described earlier, using pre-digestion for 30 min (Damgaard et al., 2015). Two aDNA extractions were performed per subsample, using a 96 well format, combining 150 µl of demineralized material with 1.5 ml binding buffer (500 ml Qiagen PB, supplemented with 15 ml Sodium acetate 3M, and 1.25 ml 5M NaCl, phenol red, adjusted to pH=5) and 10 µl of paramagnetic beads (G-Bioscience, #786-915) for 15 minutes (Rohland et al., 2018). Pelleted beads were washed twice in 450 µl and 100 µl 80% ethanol + 20% 10mM Tris-HCl, respectively, and eluted in 10 mM Tris-HCl + 0.05% Tween-20. From each subsample one extract (35 µl) was incubated with 10 µl USER enzyme (NEB #M5505) for 3h at 37°. DNA shotgun sequencing libraries were prepared in 96-well format essentially as described elsewhere (Meyer and Kircher 2010), using a small (25ul) or large (50ul) total reaction volume for non-USER and USER-treated extracts, respectively, including 21.25 µl or 42.5 µl DNA template. Clean-up procedures after end-repair and adapter-ligation were performed with 10 µl of paramagnetic beads (G-bioscience) in 10 volumes of the binding buffer described above. The requirement for PCR amplification was evaluated by qPCR using 1µl of pre-amplified library. Indexing PCR, using 8-bp unique dual indexing (Illumina TruSeq UDI0001-0096) in 50 or 100 µl reaction volumes, with KAPA HiFi HotStart Uracil+ (KapaBiosystems #KR0413) according to manufacturer's recommendations, with typically 14 amplification cycles. Final purification of libraries was performed using a 1:1.6 ratio of library to HighPrep™ PCR beads (MagBio, #AC-60250). Length distribution and concentration of individual purified libraries was controlled using the Fragment Analyzer (High Sensitivity kit). Libraries were pooled equimolar before sequencing. Sequencing was performed on Illumina NovaSeq6000 at the GeoGenetics Sequencing Core, Copenhagen, using S4 200 cycles kits version 1.5.
Timing and spatial scale	Samples from three cemeteries in Denmark were sequenced. The urban medieval churchyard of Our Lady (Vor Frue) and associated building structures were excavated by Aalborg Historiske Museum/Nordjyske Museer between 2011 to 2013. The cemetery Ahlgade 15-17 is an urban cemetery in the center of Holbæk, north of the main street of Ahlgade and adjacent to the fjord and the harbour. It was excavated in 1985 to 1986 by Museum Vestsjælland, previously called Museet for Holbæk og Omegn. The cemetery Tjaerby was excavated by Kulturhistorisk Museum Randers in 1998 to 2010.<br><br>More details are available in the SI.
Data exclusions	Low coverage and related samples were excluded.
Reproducibility	Bootstrap resampling was used for the ARS and WAP analyses. HTRX was trained out-of-sample.
Randomization	No groupings used. Covariates used were age, sex, first 20 PCs for analyses involving the UK Biobank.
Blinding	Blinding not possible.

Did the study involve field work? ☐ Yes ☒ No

Reporting for specific materials, systems and methods

We require information from authors about some types of materials, experimental systems and methods used in many studies. Here, indicate whether each material, system or method listed is relevant to your study. If you are not sure if a list item applies to your research, read the appropriate section before selecting a response.

Materials & experimental systems

n/a	Involved in the study	
☒	☐	Antibodies
☒	☐	Eukaryotic cell lines
☐	☒	Palaeontology and archaeology
☒	☐	Animals and other organisms
☒	☐	Clinical data
☒	☐	Dual use research of concern
☒	☐	Plants

Methods

n/a	Involved in the study	
☒	☐	ChIP-seq
☒	☐	Flow cytometry
☒	☐	MRI-based neuroimaging

Palaeontology and Archaeology

Specimen provenance	The urban medieval churchyard of Our Lady (Vor Frue) and associated building structures were excavated by Aalborg Historiske

Specimen provenance	Museum/Nordjyske Museer between 2011 to 2013. The churchyard belonged to the church and convent of Our Lady and is located in the eastern part of the medieval town of Aalborg. Approximately 900 graves were recovered of which 272 could be sampled for DNA analysis. The churchyard was excavated in connection with a large sewerage project, and only parts of the churchyard was exhumed.<br><br>The cemetery Ahlgade 15-17 is an urban cemetery in the center of Holbæk, north of the main street of Ahlgade and adjacent to the fjord and the harbour. It was excavated in 1985 to 1986 by Museum Vestsjælland, previously called Museet for Holbæk og Omegn.<br><br>The cemetery belonged to the former parish church of St. Nicolai and date from the late 12th century to 1573 when the church was abandoned. However, the cemetery is thought to have been taken out of use shortly after the reformation in 1536.<br><br>Tjaerby: Rural cemetery ca. 5 km east of Randers on the north side of Randers fjord. It was excavated by Kulturhistorisk Museum Randers in 1998 to 2010. The excavation area revealed a stone church, and a cemetery containing ca. 1200 graves from which 351 individuals were sampled for DNA analysis in this project. The cemetery dates to ca. 1050 to late 1536, but skeletal remains were only preserved from graves dating after 1200. Remnants of a farmhouse and a wooden church predating the cemetery (900-1100) were also recovered1,2. The surrounding area consisted of forest and meadows.
Specimen deposition	Specimens are with the museums described above.
Dating methods	Described in 'sample provenance'. Dating was either C-14 or by archaeological context. Dates are reported in SI.

☒ Tick this box to confirm that the raw and calibrated dates are available in the paper or in Supplementary Information.

Ethics oversight	No ethical approval required.

Note that full information on the approval of the study protocol must also be provided in the manuscript.
